# Increasing incidence of mycotoxicosis in South-Eastern Germany: a comprehensive analysis of mushroom poisonings at a University Medical Center

**DOI:** 10.1186/s12876-024-03550-y

**Published:** 2024-12-17

**Authors:** Petra Stöckert, Sophia Rusch, Sophie Schlosser-Hupf, Alexander Mehrl, Katharina Zimmermann, Vlad Pavel, Patricia Mester, Andreas M. Brosig, Tobias Schilling, Martina Müller, Stephan Schmid

**Affiliations:** 1 Department of Internal Medicine I, Gastroenterology, Hepatology, Endocrinology, Rheumatology, and Infectious Diseases, University Hospital Regensburg, Franz-Josef-Strauß-Allee 11, 93053 Regensburg, Germany; 2 Institute of Clinical Chemistry and Laboratory Medicine, Transfusion Medicine, University Hospital Regensburg, Regensburg, Germany; 3 Department of Interdisciplinary Acute, Emergency and Intensive Care Medicine (DIANI), Klinikum Stuttgart, Stuttgart, Germany

**Keywords:** Mushroom poisoning, Liver failure, Therapeutic plasma exchange, Climate change, Treatment outcomes, Intensive care medicine

## Abstract

**Background:**

Mushrooms, an integral component of human diets, range from esteemed delicacies to potentially lethal toxins. The risk of severe poisoning from misidentified species, poses a significant challenge. For clinicians, recognizing mushroom poisoning amidst nonspecific symptoms and determining the specific mushroom ingested are critical yet complex tasks. Additionally, climate change affects the distribution and proliferation of mushroom species, potentially heightening the risk of exposure to toxic varieties. The identification of mushroom intoxication is critical for appropriate treatment. Poisoning with highly toxic species, such as *Amanita phalloides* (death cap), can result in acute liver and kidney failure. Considering the limited therapeutic options currently available for acute liver failure, we investigated the application of plasmapheresis, a procedure involving the replacement of the patient's plasma with donor plasma, as a potential intervention to improve clinical outcomes in severe cases of mushroom poisoning.

**Methods:**

This study aimed to assess the trends and treatment outcomes of mushroom poisoning cases from 2005 to 2022, with a particular focus on the number of incidents and the potential impacts of climate change. We undertook a retrospective monocentric cohort study, evaluating 43 patients with mushroom poisoning. The study focused on identifying the variety of mushrooms involved, including psychotropic, spoiled, inedible, or toxic species, and closely examined patients with elevated transaminases indicative for liver damage. To assess clinical outcomes, we evaluated several aspects, including hepatic encephalopathy and other symptoms. Additionally, we monitored blood analysis results through serial measurements, including transaminases, bilirubin, INR, and creatinine levels. Furthermore, we explored the impact of climate changes on the incidence of mushroom poisoning.

**Results:**

While the incidence of mushroom poisonings remained relatively stable during the first eight years of the study period, it nearly doubled over the past nine years. Nine distinct mushroom types were documented. The study showed no change in season patterns of mushroom poisonings. In cases of severe liver damage accompanied by coagulopathy, plasmapheresis was utilized to replace deficient clotting factors and mitigate the inflammatory response. This intervention proved effective in stabilizing coagulation parameters, such as the international normalized ratio (INR) Plasmapheresis was performed until the INR reached stable levels, preventing the occurrence of severe bleeding complications. In instances where liver failure was deemed irreversible, plasmapheresis functioned as a bridging therapy to manage bleeding risks and to stabilize the patient while awaiting liver transplantation.

**Conclusion:**

The findings underscore the need for heightened awareness among healthcare professionals regarding mushroom poisoning and emphasize the importance of considering climate change as a factor that may alter mushroom distribution and toxicity. Additionally, this study highlights the potential of plasmapheresis in managing severe cases.

**Supplementary Information:**

The online version contains supplementary material available at 10.1186/s12876-024-03550-y.

## Background

Mushrooms, classified as eukaryotic organisms [1], have been integral to human nutrition [2] and medicinal practices since ancient times [3], valued for their culinary attributes and therapeutic properties [4]. Globally, an estimated 100,000 species of mushrooms exist, with their prevalence varying according to seasonal, meteorological, and regional factors [5]. The temporal onset of symptoms post-consumption is a critical factor, with early manifestation (< 6 h) typically correlating with more favorable prognoses than delayed presentations.

Initial symptoms of toxicity often involve the gastrointestinal system, manifesting as abdominal pain, diarrhea, and vomiting, potentially escalating to multi-organ failure, particularly affecting liver and kidneys [6]. Mushroom-induced syndromes can be categorized into six distinct groups: cytotoxic, neurotoxic, myotoxic, metabolic/endocrine poisonings, gastrointestinal irritants, and miscellaneous adverse reactions [6]. The most perilous intoxication in Europe is attributed to the consumption of the death cap (Amanita phalloides) [7]. This mushroom contains cyclopeptide compounds, notably Amatoxins, which are heat-resistant [8] bicyclic octapeptides [9]. The lethal dose for adults is approximately 0.1 mg/kg body weight, underscoring the high risk associated with even minimal consumption [10].

Intoxication from A. phalloides unfolds in three stages, beginning with severe gastrointestinal distress (abdominal pain, nausea, vomiting, and diarrhea) following a latency of 6–24 h [11]. This is succeeded, within 12–48 h, by cytolytic hepatitis marked by elevated liver enzymes and hepatic apoptosis [12]. Progressing liver and renal failure typically develop after 24–72 h, accompanied by coagulopathy, encephalopathy, and nephropathy. The primary toxins involved are α-Amanitin and Phallotoxines [13], the former being resistant to digestive enzymes and readily absorbed [14], inhibiting the RNA-Polymerase II subunit RPB1 in a p53- and caspase-3-dependent manner, leading to halted DNA-to-mRNA transcription, and consequently, apoptosis and necrosis in hepatocytes and kidneys [15]. The recirculation of α-Amanitin in the enterohepatic system prolongs its presence in the body [16].

**Fig. 1:**
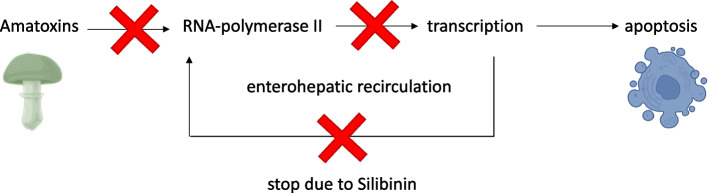
Impact of Amatoxins on Hepatocytes: Amatoxins specifically inhibit RNA polymerase II, thereby obstructing transcription and leading to apoptosis predominantly in hepatocytes. Silibinin acts to block the absorption of amatoxins by hepatocytes and disrupts the enterohepatic recirculation of these toxins

Established therapies include N-Acetylcysteine, Penicillin G, and Silibinin [17]. Silibinin, derived from the milk thistle (Silybum marianum), impedes the OATP1B3 transporter, thus blocking amanitin uptake into hepatocytes, and simultaneously promotes structural protein regeneration and enhances oxidative stress resistance [18] (Fig. 1). Penicillin G, acting as a competitive substrate for OATP1B3, also inhibits amanitin uptake [14]. N-Acetylcysteine, known for its use in acetaminophen poisoning, leverages its antioxidative properties in combating death cap (*Amanita phalloides*) intoxication. In the liver, it metabolizes to cysteine, which assists in glutathione synthesis, thereby mitigating apoptosis and inflammation. Mouse model studies have shown a decrease in leukocyte infiltration in hepatocytes following N-Acetylcysteine administration [19]. Current consensus suggests that Silibinin, either alone or in combination with N-Acetylcysteine, is the most effective treatment modality [9].

## Methods

### Study design and participant demographics

This research, conducted by the Department of Internal Medicine I at the University Hospital of Regensburg, Regensburg, Bavaria, Germany, encompassed a retrospective monocentric cohort analysis. Adherence to ethical standards was ensured by conducting the study in alignment with the Declaration of Helsinki, and it received approval from the Institutional Review Board of the University Hospital Regensburg (approval code: 24–3691-104).

The cohort comprised 43 individuals who received medical care either in the Intensive Care Unit (ICU) or the general ward, diagnosed with mycotoxicosis. We included all patients who required medical care after consuming mushrooms.Diagnostic procedures involved consulting a mycology expert for examining any available mushroom remnants or photographs thereof. In cases where physical specimens were unobtainable, descriptive and locational information was relayed to the expert.

### Clinical data and outcome measures

Upon admission, the Sequential Organ Failure Assessment (SOFA) score was recorded, along with the duration of hospital and ICU stays, to assess the intensity of medical care required for each patient. Patient demographics (age, gender, nationality), symptom onset, specific mushroom type, and the need for dialysis or invasive ventilation were meticulously documented from medical records to identify any potential correlations among these variables. Furthermore, laboratory values, including transaminases (U/L), International Normalized Ratio (INR), creatinine (mg/dL), serum urea (mg/dL), and bilirubin (mg/dL), were recorded both at admission and at their peak levels to assess liver function and organ failure resulting from mushroom intoxication. The INR was specifically used to evaluate the synthetic function of the liver. In cases of coagulopathy, plasmapheresis was utilized either as a transitional therapy to recovery or as a preparatory step for transplantation. Plasma exchanges were executed using a Spectra Optia cell separator (Terumo BCT, Lakewood, CO, USA), employing solely therapeutic plasma for exchange and Acid-Citrate-Dextrose (ACD-A) for anticoagulation, aiming for a minimum of 1.5 times the patient's total plasma volume exchange. The decision to initiate plasmapheresis was based on the INR value. In cases of acute liver failure with coagulopathy, plasmapheresis was performed daily until the INR stabilized.

To correlate the documented cases with external factors, particularly weather conditions, we used data from the German Weather Service (Offenbach, Germany), focusing on daily average temperature and rainfall.

### Data acquisition and statistical analysis

Data were sourced from SAP Software (Walldorf, Germany) and MetaVision (iMDsoft, Düsseldorf, Germany), which are used for documentation in our hospital. Statistical analyses were performed using Microsoft Excel (Munich, Germany) and IBM SPSS Statistics (Munich, Germany). Quantitative data are presented as median or mean ± standard deviation and range, while categorical variables are expressed as absolute numbers and percentages.

## Results

### Patient cohort and demographics

From 2005 to 2022, 43 patients diagnosed with mycotoxicosis were treated at the Department of Internal Medicine I at the University Hospital of Regensburg. Among these, 58.1% (*n* = 25) were male, and 41.9% (*n* = 18) were female. Outpatient treatment was provided to 14% (*n* = 6) of the patients, while the remaining 86% (*n* = 37) required hospitalization. Of the hospitalized group, 67.6% (*n* = 25) were admitted to the ICU, and 32.4% (*n* = 12) were treated in the general ward. The median age of the patients was 52 years (SD 18.4) (Table 1).

**Table 1:** Characterization of the study population. Epidemiological and clinical characteristics of the study population

Characteristics of the study population	
**Age (years):** median ± SD [range]	52 ± 18.4
**Sex:** n(%)	
Female (%)	18 (41.9)
Male (%)	25 (58.1)
**Hospitalization:** n(%)	37 (86%)
**Admission to ICU:** n (%)	25 (67.6%)
**General ward:** n (%)	12 (32.4%)
**ICU stay (days):** Median ± SD [range]	2 ± 2.3
**Overall hospital stay (days):** Median ± SD [range]	4.9 (± 5.6)
**SOFA—Score on admission**	4 (± 4.7)
**Death**	1
**Liver Transplantation**	1
**Acute kidney injury**	
Yes	10
No	33
**Dialysis**	
Yes	2
No	41
**Transaminases**	
Elevated ALT (= GPT)	18
Additional elevated AST (GOT)	11
**Mechanical ventilation**	
Yes	3
No	40

### Seasonal trends and mushroom varieties

**Fig. 2:**
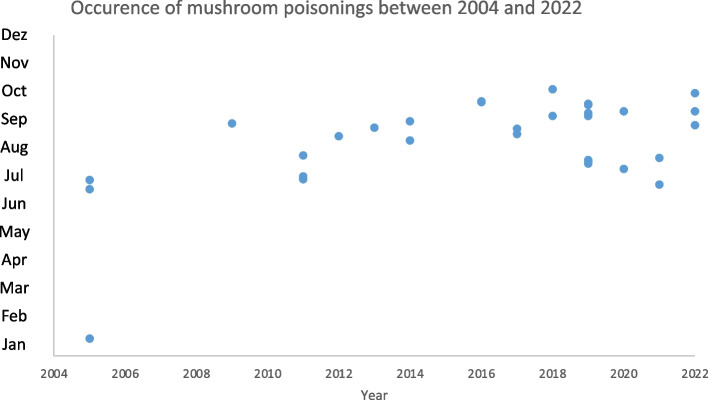
Occurrence of Mushroom Poisonings, 2005–2022: This timeline illustrates the trend in mushroom poisoning incidents over the study period. Although there was a noticeable increase in the number of poisonings, the distribution of cases throughout the year remained consistent

**Fig. 3:**
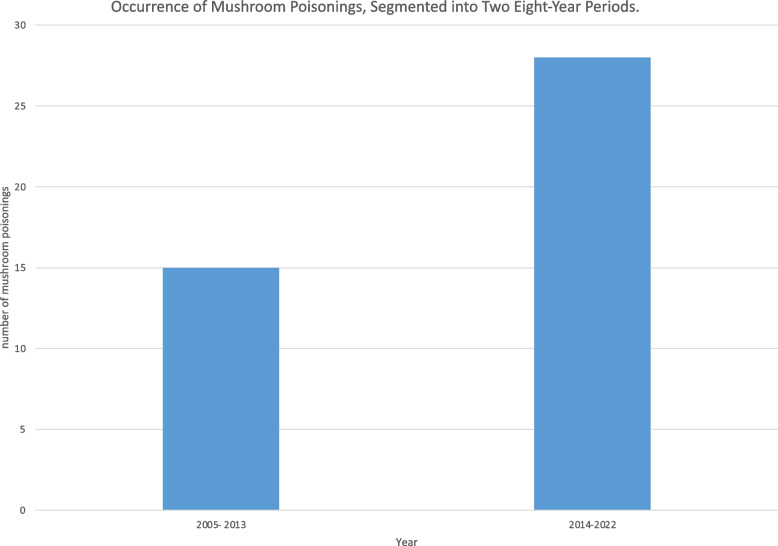
Occurrence of Mushroom Poisonings, 2005–2022, Divided into Two Periods: The graph details mushroom poisoning cases over two distinct nine-year periods. During the initial period, 15 patients were documented. This figure rose to 28 in the subsequent nine years, illustrating a marked increase in the incidence of mushroom poisonings

**Fig. 4:**
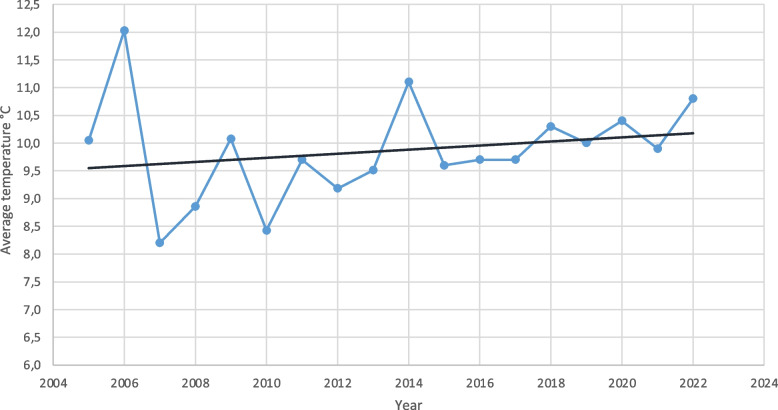
Rising Autumn Temperatures, 2005–2022. This figure illustrates the gradual increase in average autumn temperatures over the study period, from 9.5 °C in 2005 to 10.2 °C in 2022

The study also investigated whether climate change has precipitated an earlier annual occurrence of mushroom poisonings. By aggregating incidents where multiple individuals consumed mushrooms into single cases, we could not observe an earlier annual occurrence due to climate change (Fig. 2). Poisonings occurred predominantly between August and November. Notably, there was an increase in poisoning cases, from 15 in the first 9 years to 28 in the subsequent 9 years (Fig. 3). Over the period examined, the average autumn temperature in Germany increased by 0.7 degrees Celsius, indicative of climate change impacts (Fig. 4).

**Fig. 5:**
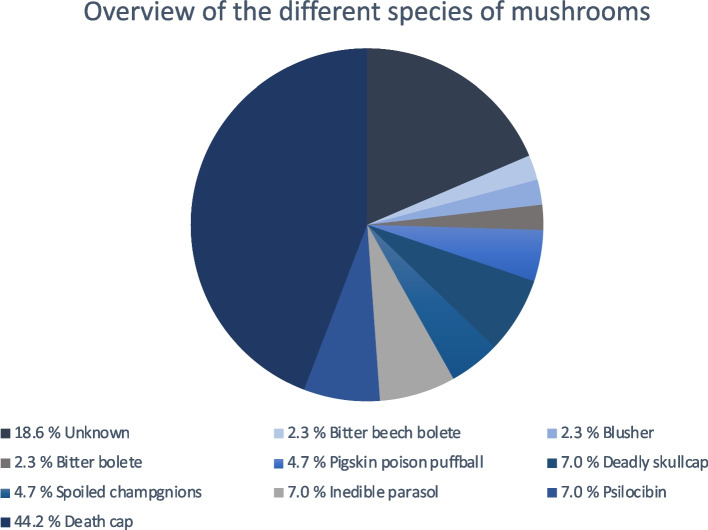
Overview of intoxications with different mushrooms species. Most frequently the intoxications are caused by death cap (Amanita phalloides)

Nine different mushroom species were implicated in the cases, with death cap (*Amanita phalloides*) poisonings comprising the majority (44.2%, n = 19). Other cases included intoxications with psychotropic mushrooms (*Psilocybe semilanceata*) (7%), inedible parasols (*Chlorophyllum venenatum*) (7%), spoiled champignons (*Agaricus* species) (4.7%), deadly skullcaps (*Galerina marginata*) containing amatoxins (7%), and pigskin poison puffballs (*Scleroderma citrinum*) (4.7%). Less common were cases involving bitter boletes (*Tylopilus felleus*), blushers (*Amanita rubescens*), and bitter beech boletes (*Caloboletus calopus*), each accounting for 2.3% of cases (Fig. 5).

### Treatment modalities and outcomes

Outpatient cases typically involved the consumption of psilocybin mushrooms (*Psilocybe* species), champignons (*Agaricus* species), and bitter boletes (*Tylopilus felleus*). One patient who consumed spoiled champignons required endoscopic intervention and proton pump inhibitors due to Mallory-Weiss lesions.

Suspected death cap (*Amanita phalloides*) ingestions were promptly treated with intravenous Silibinin (20 mg/kg per day, divided into 4 doses over two hours each, until transaminase normalization or a maximum of five days). In 41,9% (18/43) cases, Silibinin was the sole therapy. Additional treatments included combinations of Silibinin with N-Acetylcysteine, activated charcoal, and Rifaximin. In situations where Silibinin was initially unavailable, Penicillin G was administered until patient transfer to our department, with N-Acetylcysteine added in two cases. Three patients exhibited highly elevated transaminase levels and significantly reduced metabolic and synthetic liver function. Among these, one patient (2.3%) died from liver failure, another received a liver transplantation, and one patient recovered spontaneously (Table 1). Psychotropic mushroom (*Psilocybe semilanceata*) and Psilocybin intoxications were managed with Lorazepam as needed, and activated charcoal was used in two instances. Eight patients (8,6%) with mild symptoms required no therapy.

### Clinical presentations and laboratory findings

86% (37/43) of patients presented with gastrointestinal symptoms, and one experienced cramps, dizziness, and headache. Three severe cases (7%) involved reduced Glasgow Coma Scale (GCS) scores, averaging 7.3, necessitating intubation. Elevated SOFA scores were observed in 25,6% (11/43) patients, ranging from 1–16, with an average of 4 (± 4.7).

### Liver and renal function analysis

41,9% (18/43) of patients presented with elevated ALT levels, 11 (61%) of whom also had elevated AST (Suppl.1). Most of these cases involved suspected death cap (*Amanita phalloides*) intoxication. The mean ALT upon hospital presentation was 1951.2 U/l (SD ± 1411.51), and AST was 1612.6 U/l (SD ± 1653.1). Bilirubin and INR levels were also elevated. An increase in serum creatinine was observed, with two cases requiring dialysis.

### Plasmapheresis and coagulation failure

Plasmapheresis was performed four times in one patient as a bridge to liver transplantation. The procedure involved multiple therapeutic plasma exchanges to manage coagulation failure. Another patient, presenting with coagulation failure, underwent two successful plasmapheresis sessions, stabilizing coagulation parameters over time. Plasmapheresis was performed based on the INR value. If the INR showed a marked increase, plasmapheresis was repeated the following day until the INR stabilized.

### Treatment outcomes and criteria assessment

**Fig. 6:**
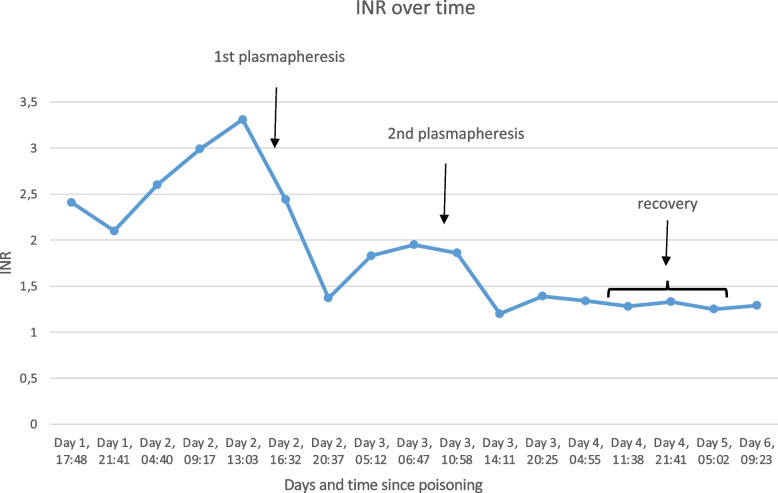
INR Course Over Time in a Patient with Severe Liver Injury. This graph displays the temporal progression of the International Normalized Ratio (INR) for a patient following severe liver injury. After two sessions of plasmapheresis aimed at preventing severe bleeding complications, the INR stabilized. Plasmapheresis proved to be an effective intervention, serving as a bridge to recovery or preparation for liver transplantation

Despite the severity of some cases, including a fatality and a need for transplantation, King's College Criteria were not met in any patient at the time of initial presentation. Nevertheless, one patient required liver transplantation. In one case with leading koagulation failure plasmapheresis was used as bridge to recovery. The dynamics of INR over the treatment period of this patient are depicted in Fig. 6.

## Discussion

This is the first systematic analyses of patients treated for mushroom poisoning at a Bavarian hospital. Diagnosing mycotoxicosis poses significant challenges due to the frequent absence of mushroom remnants and the lack of rapid, reliable detection methods [20–23]. In instances of suspected death cap (*Amanita phalloides*) ingestion, timely therapeutic intervention is critical to avert fatal outcomes [24]. Laboratory detection is feasible in specialized facilities; however, its clinical utility is limited by substantial time delays and potential for false negatives due to symptom latency. Clinical judgment, grounded in a thorough patient history and specific inquiries about wild mushroom consumption, becomes paramount, particularly for emergency department physicians who must be adept at recognizing key indicators during mushroom season.

### Climate change and seasonal trends

The study explored whether climate change has influenced the timing of mushroom poisonings since 2005. Our findings indicate no significant shift in the seasonal occurrence of these incidents [25], which predominantly take place between August and November [26, 27]. As temperatures rise, there is a corresponding increase in hydro-climatic extremes [28]. Elevated rainfall intensity and more frequent flood maxima [29], coupled with warmer conditions, are conducive to fungal proliferation in autumn. The average rainfall in recent years has frequently exceeded the annual averages from many years ago [30]. Consequently, regions previously too cold or dry are now witnessing the emergence of various new fungal species. This shift in fungal occurrence enhances the likelihood of species misidentification, thereby increasing the risk of accidental intoxications [31]. In 2015, a correlation between rising average temperatures and mushroom poisonings was identified in Hunan Province [32]. Another group observed a correlation between meteorological factors and mushroom poisonings as well [33].

### Rising incidence of mushroom poisonings

An increasing number in mushroom poisonings over the past nine years was observed. This rise is probably attributed to a greater public inclination towards nature-based leisure activities, including mushroom foraging [34]. The proliferation of digital resources and identification apps may have paradoxically fostered a false sense of security, reducing the hesitation to gather and consume wild mushrooms [35]. This underscores the need for continued public education to highlight the risks of potentially fatal misidentifications.

### Identifying toxic versus inedible mushrooms

Our data documents nine different mushroom types, illustrating the diagnostic diversity. Two primary challenges exist: accurate mushroom identification and differentiation between inedible and poisonous varieties [36]. Mushroom collectors believe often that they can identify the mushroom correctly [37]. However, misidentification is the most common cause of intoxications [38, 39]. The former often requires consulting local mycological experts, while the latter is complicated by similar early symptoms across various species. Apart from psychotropic mushrooms and Psilocybin [40], all documented types induced gastrointestinal symptoms, a common early sign of death cap (*Amanita phalloides*) ingestion [41, 42]. Therefore, patients presenting with such symptoms during mushroom season should be questioned about mushroom consumption. Laboratory parameters like transaminases and creatinine are critical for differential diagnosis. The study generally did not pursue toxicological verification in biological samples due to the limited availability of specific laboratory tests for various fungal toxins.

### Plasmapheresis in clinical management

The non-transplant treatment options for patients with acute liver failure are very limited. Therapeutic plasma exchange is the only treatment shown to improve survival rates in these patients [43]. In cases of liver failure leading to coagulopathy, plasmapheresis has proven effective in mitigating severe bleeding risks [44–46]. A study on children with acute liver failure demonstrated an improvement in biochemical profiles. It showed that initiating plasmapheresis in the early stages of acute liver failure reduces the acute inflammatory response and can increase the likelihood of recovery [47, 48]. An elimination of the poison by plasmapheresis is not possible [49, 50]. We observed improved liver function and normalized coagulation parameters following plasmapheresis, demonstrating its utility as a "bridge to recovery” [51, 52] (Fig. 5). Additionally, in cases where liver transplantation is imminent, plasmapheresis can be instrumental in managing coagulation failure and maintaining patient stability, serving as a bridge to recovery or as a temporizing measure until transplantation becomes possible [24, 53–55].

## Study limitations

This research represents a retrospective analysis from a single center, which may limit the generalizability of its findings. Additionally, the precise identification of mushroom species often poses significant challenges. Moreover, establishing a direct causal link to climate change remains difficult due to the complexity of contributing factors. Given the relatively small number of cases and the considerable diversity of mushroom species involved, the current findings require further validation through additional studies to ensure robustness and generalizability.

## Conclusions

Mushroom poisonings can have devastating consequences. As climate change potentially extends the traditional peak mushroom seasons, physicians must be particularly vigilant. Due to climate change and an increase in nature-based leisure activities, the number of mushroom poisonings is rising. Accurate identification of the consumed mushroom is essential for initiating the correct specific therapy. Prompt initiation of treatments like Silibinin and N-Acetylcysteine is crucial in suspected death cap (*Amanita phalloides*) poisonings. Patients with significant liver enzyme elevation should be referred to liver transplantation centers. Our study suggests that plasmapheresis can serve as an effective bridge to recovery, potentially eliminating the need for liver transplantation in patients with acute liver failure and associated coagulopathy.

## Supplementary Information

Supplementary Material 1.

## Data Availability

The datasets used and/or analysed during the current study are available from the corresponding author on reasonable request.

## Abbreviations

ACD-A: Acid-citrate-dextrose solution A
ALT: Alanine aminotransferase
AST: Aspartate aminotransferase
GCS: Glasgow coma scale
GOT: Glutamate–oxaloacetate transaminase
GPT: Glutamate-pyruvate transaminase
ICU: Intensive care unit
INR: International normalized ratio
OATP1B3: Organic anion transporting polypeptide 1B3
RNA: Ribonucleic acid
SD: Standard deviation
SOFA: Sequential organ failure assessment
