# Supplementary material for: Increasing incidence of mycotoxicosis in South-Eastern Germany: a comprehensive analysis of mushroom poisonings at a University Medical Center
Source: BMC Gastroenterol. 2024 Dec 17;24:450. doi: 10.1186/s12876-024-03550-y (PMC11653814; doi:10.1186/s12876-024-03550-y)
Supplement: Supplementary file 1 — Supplementary Material 1. [file 12876_2024_3550_MOESM1_ESM.pdf]

	AST A (U/l)	AST max (U/l)	AST D (U/l)	ALT A (U/l)	ALT max (U/l)	ALT D (U/l)	Bilirubin A (mg/dl)	Bilirubin max (mg/dl)	Bilirubin D (mg/dl)	INR A	INR max	INR D
**Pat. 1**	248	248	79	2327	2327	116	4.6	10.6	10.6	2.46	2.49	1.72
**Pat. 2**	2543	4825	29	2857	4275	26	3.1	8.8	1.1	2.41	3.31	0.95
**Pat. 3**	628	628	83	1934	1934	351	4.1	11.2	5.8	2.06	2.06	1.08
**Pat. 4**	609	609	37	977	1116	209	0.8	1.3	1.1	1.07	1.08	1.03
**Pat. 5**	235	235	21	416	416	88	0.6	0.6	0.5	0.97	1.00	0.86
**Pat. 6**	5277	5388	28	4964	5345	390	5.0	11.4	6.8	4.03	5.00	1.59
**Pat. 7**	295	375	30	187	313	133	0.8	1.0	0.6	0.99	0.96	1.09
**Pat. 8**	798	798	38	657	657	210	0.5	0.6	0.3	1.03	1.03	0.86
**Pat. 9**	940	2960	1039	1008	6624	4788	2.8	6.6	6.3	1.64	2.60	1.26
**Pat. 10**	1887	1887	57	3515	3515	424	1.3	1.3	0.7	1.27	1.27	0.99
**Pat. 11**	4278	5190	112	2621	3677	262	0.8	1.6	0.5	1.12	1.21	1.04

Supplement 1: Course of AST, ALT, Bilirubin, and INR

Each parameter is documented at the time of admission (A), discharge (D), and at its peak value (max).
